# The True Physician

**Published:** 1861-07

**Authors:** 


THE TRUE PHYSICIAN.—Dr. JOHN WATSON'S Anniversary Discourse. 1860.

Delivered before the New York Academy of Medicine, November 7, 1860, and published by its order. Distinguished for its elegance and scholarly polish, it is yet no mere holiday array of fine-sounding words; but an earnest, practical appeal, high toned and liberal, for the dignity and elevation of our professional standard. It is sufficient praise to write, that it is worthy of its eminent author—the President of the New York Academy of Medicine.

